# Supplementary material for: An examination of the association between early initiation of substance use and interrelated multilevel risk and protective factors among adolescents
Source: PLoS One. 2019 Dec 11;14(12):e0225384. doi: 10.1371/journal.pone.0225384 (PMC6905657; doi:10.1371/journal.pone.0225384)
Supplement: Attachment — Submitted filename: Response to Reviewers.docx [file pone.0225384.s003.docx]

Response letter

Reviewer #1

Introduction
1. The authors indicate that risk factors will be abbreviated as “RF” and “FP”, but later use the full phrase (risk factors, protective factors) and “PF” throughout the manuscript. Please correct for consistency.

*R. These abbreviations were included along the document.*

2. In the first paragraph, the sentence, “MCA is able to capture multiple associations among interdependent factors, without making assumptions about functional forms (linear or otherwise)” needs a citation to support this claim.

*R. We included the citation.*

3. In the Introduction, and throughout the rest of the manuscript, sentence structure is at times awkward and confusing to read (e.g., the second sentence of the second paragraph starting with “Kessler et al….” and the fifth sentence of the second paragraph starting with “Chikritzhs…”). Having a native English speaker proofread the manuscript would be helpful in this regard. Also, please use decimal points rather than commas for the numbers you report in this study.

*R. We had the new version of the paper copyedited by a professional native english speaker.*

4. The section under “Risk and Protective factors associated with age of initiation” has several vague descriptions of prior findings that need additional specificity. For instance, the authors say “Kaplow et al. identified that children [childhood?] personal factors and parental patterns of drug consumption contribute to early initiation of drug use.” What are the “personal factors” in children that the authors are referring to? Personal factors could many mean different things (e.g., externalizing or internalizing behaviors, temperament, ADHD). Later in this section, the authors refer to “parental modeling” as a predictor of delayed alcohol initiation. It is unclear what behavior(s) the parents were modeling.

*R. We clarified the information in the introduction section of the paper by making more detailed reference to the cited works .*

Methods

1. The description on how the survey was implemented should be moved to the beginning of the Methods section where research procedures are discussed.

*R. We moved the description of survey implementation to the first paragraph of the methods section.*

2. What were the response options for each of the 17 risk and protective factors? In the supplement it appears as though the response scale ranges from 1-4, but it is unclear what each of these numbers equate to in relation to scale.

*R. A description of the response options to assess RF and PF was included in the methods section.*

Results/Discussion

1. Was there any missing data across the variables examined in this study? If so, the authors need to report how they accounted for missing data in their analyses.

*R. The information was collected through an electronic platform that required all answers to be recorded in order to allow participants to progress along the instrument. Therefore, there was no missing data. We included this clarification in the methods section.*

2. Since this study consisted of a cross-sectional sample of participants ages 12-19, mean age of 14, it is problematic to categorize substance use initiation as early, middle, and late. This is because the younger participants in this study may be misclassified. For example, a 12 year old in the sample who reported no use at the time of the assessment may begin use in the future by age 13 or 14 and therefore actually be an early initiator instead of a non-user. This limitation of the study needs to be addressed in the discussion section.

*R. Thanks for this observation. We included a comment about this limitation in the discussion and potential usefulness of future research that test our results using a longitudinal design. We also extended our arguments for the categorization used.*

3. In the last section of the Results, the authors categorize certain factors as RF or PF. However, it seems like each RF could be a PF if the direction of effects were in the opposite direction. For example, “family conflict” is categorized as a RF. The authors should specify that high family conflict was a risk factor, because low family conflict could be a protective factor. Please add the direction of effects in this section.

*R. According to Hawkins, Catalano and Miller (1992) RFs and PFs constitute single-directional constructs, whose absence do not exert an effect in the opposite direction. For instance, the presence of family conflict is a RF but its absence is not a protective factor. Thus, each factor has only one implicit direction of the effect, depending on its risky or protective nature. This is the quote from the Catalano and Miller work: “Though some have viewed protective factors simply as the opposite of those variables identified as risk factors (Labouvie & McGee, 1986), this conception does not appear particularly useful. Designating two distinct constructs (e.g., risk and protective factors) to distinguish extreme levels of a single variable bearing a linear relationship to drug abuse adds little. It is not necessary to postulate protective factors if better outcomes are observed in those not exposed to risk. On the other hand, if protective factors are viewed as sources of differences in response to a given amount of exposure to risk, the construct stimulates attention to nonlinear and interactive relationships among risk and protective factors” (p. 86).*

Reviewer #2

1. Modify the title in order to mention that this analysis was performed in a Colombian youth sample.

*R. Due to the fact that substance abuse behaviors seem to operate in a similar way across different cultures, we considered that it is not necessary to specify in the title the Country in which the study was developed. This information is specifically mentioned in the paper.*

2. A section that explains potential interdependence between factors could be included;

*R. We discuss in further detail theories that highlight the relevance of interdependence between RF and PF in the introduction to improve the positioning of the paper.*

3. Also, no mention was made about the Colombian evidence in the topic (if existed). The need of evidence in the field for Colombia should also be mentioned; Add references that indicate this potential factor interdependence.

*R. We included in the discussion, previous research conducted with Colombian samples about RF, PF and age of onset.*

4. Is not clear if the used survey was just the Spanish version of the CTC youth survey or was the adapted version of the CTC-Youth survey for Colombia. If the Colombian version was used, the characteristics of the local adapted survey should be mentioned and referenced in order to provide the reader with information about the survey characteristics and its initial internal consistency –Cronbach's alpha Coefficient reported in 2015 (Mejía-Trujillo, Pérez-Gómez y Reyes-Rodríguez, 2015).

*R. In the methods section, we mentioned that we used the Spanish version of the CTC-Youth survey (Arthur, Hawkins, Pollard, Catalano & Baglioni). We did not used the CQC Colombian version. However, we included the alphas for each RF and PF for internal reliability of the CTC for the study sample.*

5. The multivariate regression model that will serve as comparison was not specified. The fitted equation should be explained in order to understand the model estimations that will be contrasted with the MCA.

*R. The regression equation was included in the appropriate place.*

6. Descriptive statistics from table 1 are usually part of the methods section where the used instrument is described.

*R. Given that the descriptive statistics comes from the application of the instrument and that such results constitute the basis for the variable discretization performed in order to conduct the MCA, we thought that moving this information to the methods might affect the readability of the manuscript.*

7. In table 1, risk and protective factors could be categorized by domain to provide better a understand of the table.

*R. We modified Table 1 to provide the factors categorized as suggested by the reviewer.*

8. The distribution of participants in the three early initiation categories should be shown. This could help to understand if the age ranges created do not affect the models.

*R. The supplementary material 1 contains the information on how participants were distributed in the three categories as well as all the other categories. We included the frequencies for early initiation categories in the main text,*

9. Moreover, it was mentioned that age 15 years is considered the cut point for EI. However, in Latinametican countries such as Colombia, the mean age of initiation is lower, therefore this age cut point should be validated. For instance the age of initiation associated with alcohol disorders in Colombia could be lower compared with the countries where <15 years was identified as the risky age. A an explanation of the potential different scenarios of using 14 or 13 as the cut point should be provided or discussed.

*R. We used a compound measure of age of onset, as established in the CTC - YS that included Alcohol, Cigarettes, Marijuana and Inhalants. The mean age of onset in our sample was 1,30 in our scale, which means somewhere between 16 and 17 years old. We included these details in the results section. Given these numbers, the early initiation in our sample is not that low, even only for alcohol. Hence, the frequencies of groups below 13 or 12 would be too low rendering the sensitivity analysis unreliable. Furthermore, the age of 15 is therefore consistent with global standards of risky age of onset and the data is also consistent with such cut off points. We added this comment in the results.*

10. A better interpretation of Table 2 results could be: "The application of multiple correspondence analysis showed that the total inertia explained is equal to XXX (percent of inertia: 23.6% is due to the first axis dimension 1 and 13% due to the second dimension).

*R.We made this modification in the results.*

11. A remarkable simple explanation of the figures was made. In the figures the specific percentage of variance for each dimension should appear [e.g. Dim 1 (23.6%); Dim 2 (13.0%)].

*R. All the figures are partial biplots produced from the same MCA analysis, hence the information about the dimension´s inertia are the same in all figures. We included this clarification in the results section.*

12. It was not mentioned if interactions were examined. Significant interaction effects could modified the effects sizes . Also the fitted equation was not specified: Example: Y = βO + β1RFc + β2AGEc + β3GENDERc + β4RFc*AGEc+ β5RFc*GENDERc
Moreover, a multiple logistic regression could be considered for better comparison with MCA.

*R. The regression equation was explained and included in the results section. In addition, we conducted an ordered logistic regression using the categorical three-level variable for early initiation, as used in the MCA, to check the robustness of the linear regression model. They were highly consistent. We report this in the results section and add a supplementary file with the results of the ordered logit.*

*Regarding the interactions, we did not estimate interaction terms in the regression model. This becomes clear in the equation included. Whereas it is true that including interactions in regression models modify regression coefficients (the effects of the interacted terms change from main effects to simple effects) (Hayes, 2013) we had no hypotheses about certain interactions, or moderating effects, of interest. Furthermore, estimating a model with all possible interactions between RF and PF (this is the key MCA ability) would yield (18*18)/2 = 162 additional regression terms, which is obviously impossible and impractical to analyze. We do take from this comment that future research should review specific moderating effects between RF and PF that are worth exploring. We include in the discussion a comment about the limitation of MCA to study moderation effects that can be captured through interaction terms within regressions.*

13. There was no mention about how the authors dealt with missing data.

*R. The information was collected through an electronic platform that required all answers to be recorded in order to allow participants to progress along the instrument. Therefore, there was no missing data. We included this clarification in the methods section.*

14. "Regarding specific RF and PF, our analyses highlight that the effect of perceived availability of drugs remarkably persist across different methodologies suggesting that such perception must be prioritized in health policy."- This was a good conclusion. However should be confirmed after checking for interactions in the regression model.

*R. As mentioned in the previous answer, we don´t have specific interactions to test among predictors. We agree that developing and testing hypotheses about such interactions may extend and deepen our conclusions.*

15. The manuscript did not include any limitations of the study. One limitation could be that despite this research contributes to understand these causes by assessing EI relationship with multiple RF and PF in the proximal and distal environment of the adolescent, no subgroup analysis could be done and was not discussed –due to the methods selected–.

*R. We included a section on limitations of using MCA after the first paragraph of the discussion, where the differences between MCA and regression are discussed. We emphasized the inability of MCA to assess moderating effects and the problems derived from categorization of age of onset,*
