# Supplementary material for: An examination of the association between early initiation of substance use and interrelated multilevel risk and protective factors among adolescents
Source: PLoS One. 2019 Dec 11;14(12):e0225384. doi: 10.1371/journal.pone.0225384 (PMC6905657; doi:10.1371/journal.pone.0225384)
Supplement: Attachment — Submitted filename: Response to reviewers.docx [file pone.0225384.s004.docx]

Dear editor,

It is with great pleasure that we are resubmitting the revised (R2) version of the manuscript PONE-D-19-16848 titled “*An examination of the association between early initiation of substance use and interrelated multilevel risk and protective factors among adolescents*”

These are the responses to your comments:

Comment 1. The description of the analysis in the Methods section is not sufficiently detailed. Please move all text describing the steps of the analysis and the software used for analysis into the Methods section from its current position in Results.

*R/ We adjusted the sections as requested.*

Comment 2. Please add a note to each figure caption to aid in interpreting the figure without consulting the text. It is not immediately clear what 1-2, 2-1, 3-1, etc., indicate in the figure and the figures therefore do not stand alone.

*R/ We added the requested note in each figure such that the reader can open any figure in any order.*

3. The readability of the figures could also be improved through changing font size, size of the shapes on the plot, and using more distinctive shapes that will be distinguishable in black and white as well as color.

*R/ We made our best effort to improve the figures readability by making several changes in changes, fonts, colours and styles, as much as the software offers tools to do it.*
